# Linking Geospatial and Laboratory Sciences to Define Mechanisms behind Landscape Level Drivers of Anthrax Outbreaks

**DOI:** 10.3390/ijerph16193747

**Published:** 2019-10-04

**Authors:** Michael H. Norris, Jason K. Blackburn

**Affiliations:** 1 Department of Geography, University of Florida, Gainesville, FL 32611, USA; mhnorris@ufl.edu; 2 Spatial Epidemiology and Ecology Research Lab, University of Florida, Gainesville, FL 32611, USA; 3 Emerging Pathogens Institute, University of Florida, Gainesville, FL 32611, USA

**Keywords:** anthrax, luminescence, spores, infectious disease, zoonosis, NDVI, outbreak, *Bacillus anthracis*, interdisciplinary

## Abstract

*Background*: A seasonal predictor of anthrax outbreaks is rainfall, which may be approximated by NDVI using remote sensing. How rainfall or vegetative green-up influences bacterial physiology or microecology to drive anthrax outbreaks is not known. *Methods*: Rainfall and NDVI dependency of anthrax epizootics was demonstrated with global and local phenological analysis. Growth analysis of *B. anthracis* in response to pH and calcium gradients was carried out. The influence of pH and calcium levels on expression of toxin and sporulation related proteins in broth culture models was characterized using engineered *B. anthracis* luminescent reporter strains. *Results*: Short-term bacterial growth and longer-term bacterial survival were altered by pH and calcium. These conditions also played a major role in *pagA* and *sspB* promoter-driven luminescent expression in *B. anthracis. Conclusions*: Rainfall induced cycling of pH and calcium in soils plays a plausible role in amplifying spore load and persistence in endemic anthrax zones. Observed evidence of *B. anthracis* favoring soil alkalinity and high soil calcium levels in the environment were linked to physiological conditions that promote bacterial growth, survival, toxin secretion and spore formation; illustrating the utility of bringing laboratory-based (controlled) microbiology experiments into the fold of zoonotic disease ecology.

## 1. Introduction

Environmentally-mediated pathogen transmission occurs when environmental conditions are suitable for both hosts and pathogens to survive an intersect. While pathogen survival may vary from short-term (days or weeks; e.g., *Brucella* spp.) or long-term (years or decades; e.g., *Bacillus anthracis*), disease intensity varies over space and with apparent, but poorly understood, predictability or seasonality. This may occur across diverse landscapes, but the mechanisms for increased disease intensity is poorly studied for environmentally mediated pathogens.

Anthrax, a disease recognized since ancient times, is caused by the Gram-positive spore former *Bacillus anthracis*. This same pathogen served as the model organism from which Koch’s postulates originated and bioterrorism immediately comes to mind. Naturally occurring, anthrax is a zoonosis with frequent epizootic outbreaks and spillover to humans is common and problematic in many countries [1]. Soil-borne *B. anthracis* spores are found in specific soil conditions nearly worldwide and are infectious to animals such as grazing ruminants, often resulting in high mortality. Phylogenetically, *B. anthracis* has traditionally been divided into four clades, A, B, C, and D. Group A *B. anthracis* has spread to all continents (but Antarctica). Other groups have not dispersed as widely [2]. The highest degree of genetic diversity is found in southern Africa and was hypothesized as the potential geographic origin of anthrax evolution because it represented the only location in the world (Kruger National Park; KNP) where *B. anthracis* from groups A and B coexist [3]. These strains show geographic affinities within the park, with group A localizing to the center of the park and group B to the north. Group A strains were found in soils with a mean soil pH of 6.74 and mean calcium content of 185.68 me/kg. Group B strains were found in soils with higher mean soil pH and mean calcium content, pH 7.76 and 274.14 me/kg, respectively. Interestingly, during anthrax epidemics in KNP, isolates from both groups A and B cause infection. This suggests that sources external to strain, pH, or calcium content trigger infections/epizootics; Though likely contributors to environmental persistence of the pathogen, triggers of infections/epizootics peripheral to strain, pH, or calcium content are suggested by the co-circulation of strains during outbreaks in KNP. Strain differences can still impact infectiousness, virulence, and microecology of anthrax. The external sources that trigger outbreaks are as yet underdetermined environmental signals. Spores are reported to have a half-life of approximately 100 years [4] and environmental elimination is not an easy task as evidenced by the intensive decontamination efforts on the small Scottish island of Gruinard [5]. Considering the length and tenacity of the organisms’ environmental persistence, complete eradication of *B. anthracis* from endemic areas is seemingly unachievable. 

In the soil environment, the bacterium exists as a dormant (metabolically inactive) spore, waiting for the right signals to permeate through the exosporium layer and initiate germination. Vegetative organisms are sensitive to desiccation and heat but can sporulate quickly during conditions unfavorable for growth. The spore surface, or exosporium, is coated with a glycoprotein that is involved in spore binding to environmental surfaces, generates spore hydrophobicity and affects spore germination [17,18,19]. Spores contact a host through ingestion, inhalation, or cutaneous inoculation then germinate to the vegetative form and elaborate the A_2_B-type anthrax toxins made up of protective antigen (PAG), which transports both lethal factor (LF) and edema factor (EF) into the cell cytoplasm, causing animal death by toxemia.

### 1.1. Seasonal Predictors of Anthrax Epidemics

**Figure 1:**
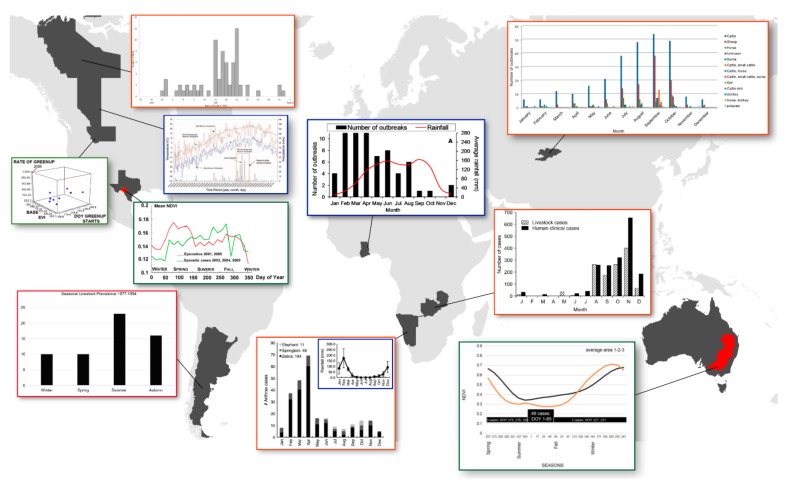
Seasonality of anthrax outbreaks is a global phenomenon. Here we illustrate clear patterns of outbreak intensity and timing associated with month (orange bounding boxes; Kyrgyzstan [6]; Northwest Territories, Canada [7]; Zambia [8]), season (red bounding box; Argentina [9]), precipitation (blue bounding boxes; Alberta, Canada [10], Ghana [11], Etosha National Park, Namibia [12]), enhanced vegetation index (green bounding box; EVI; Montana, Blackburn & Goodin, unpublished data), and normalized difference vegetation index (dark green bounding boxes; NDVI; Australia [13] and Texas [14]). Red areas in Texas and Australia are the recently defined enzootic zone [15] and re-defined anthrax belt [16], respectively.

Anthrax is often thought of as an important agent of bioterrorism. While certainly such a risk exists, anthrax most often causes disease in wildlife and livestock due to its persistence in soil around the world with spillover into proximate human populations. Globally, the disease remains a public and animal health problem, with recent increases in human disease in the Republic of Georgia illustrating the threat clearly [20]. Outbreaks still occur with frequency in the US [21], including areas where vaccination reaches livestock but not wildlife [15]. Outbreaks occur in an episodic fashion with pronounced seasonality (Figure 1) and some studies have modeled this for systems where there is no active disease control [22]. Notably, seasonality has been defined study by study, and often with different metrics, such as cases or outbreaks by month or season. Other studies have measured outbreaks against precipitation or vegetation indices. Here we compile those data in a single map to discuss patterns globally. It is estimated that 1.1 billion animals are at risk of anthrax globally and that 198.2 million *B. anthracis* Sterne livestock vaccines are administered each year with potential to impact adjacent human populations totaling 63.5 million [1]. In the high-risk anthrax belt of Australia, early spring and hot dry summers have been associated with severe livestock anthrax [23]. A close look at the annual normalized vegetation index (NDVI) trajectory, as an evaluation of seasonal environmental changes, showed that summer green-up (associated with rainfall) often occur in the weeks or months prior to the major outbreaks in epizootic years possible [13]. In Etosha National Park (ENP), Namibia, anthrax is also associated with rain events where there is a clear relationship between monthly precipitation and cases [12,24,25]. In Ghana, a bimodal climatic trend applies to areas of the most numerous and intense epizootic anthrax outbreaks [11]. Of all anthrax cases in Zambia, where anthrax is hyper-endemic, ~85% of 2108 human cases over a 10-year period occurred in the Zambezi floodplain of western Zambia [8]. Livestock cases closely mirror the human cases and primarily occur during hot-dry months after the rainy season when herding brings animals towards the dried flood plain. It is also important to note that if the only factor leading to anthrax outbreaks were early spring green-up and hot summer-associated rainfall then anthrax outbreaks would be easily predictable. Anthrax outbreaks do not occur annually and are considered episodic [22,26], possibly with many endemic years between epizootic years. The 2008 anthrax outbreak in Montana is a prime example. After nearly 50 years of no major outbreaks, a severe outbreak occurred among bison and elk, killing ~8% of the bison herd [27,28]. Outbreaks in North America are reported at lower frequency than in Africa, but still ensue near temperature maxima and following periods of locally high rainfall, as during the localized 1999 Alberta anthrax outbreak which affected seven cattle farms [10]. The authors have recently (31 July–5 August 2019) returned from an outbreak in West Texas described by long-time regional veterinarians as the worst outbreak in a lifetime. The 2019 Texas anthrax outbreak is nearly a perfect fit for the above described predictors. It encompassed at least 2000 km^2^ of ranchland and claimed numerous animal species (white tailed deer, audad, oryx, barasingha, antelope, red deer, axis, hartebeest, and gemsbok, among others). West-Texas experienced a wet spring, followed by a year’s worth of rainfall in June 2019 that preceded dry conditions with temperatures at or above 38 °C (100 °F). Indeed, in his seminal work on anthrax ecology, Van Ness linked water related geographic features with anthrax in the incubator hypothesis [29]. Although there are drawbacks to the incubator hypothesis, it aligns well with the observed cyclic weather events. Additionally, high soil moisture and organic content are conducive to bacterial survival whether in vegetative or spore form. Anthrax outbreaks occur across varying geographies and ecologies and can be perpetuated by place-specific mechanisms.

In Texas, the vegetation is diverse, containing scrub, shrubs, and trees while in the highly endemic ENP the landscape is relatively homogenous grassland. In ENP, anthrax is most frequently found in grazing animals, including zebra [24]. It has been shown that *B. anthracis* is associated with plant roots and that ruminants, such as zebra, can consume large doses of anthrax during grazing contact with the soil reservoir of *B. anthracis* [30]. The 2008 Montana outbreak affected bison and elk, both grazers, in Montana [27]. In both species, male anthrax mortality rates were much higher than females. It has been observed that males of both species have a higher frequency of inspecting carcasses than females translating to higher rates of serological exposure (Blackburn unpublished data). West Texas is a major anthrax focus in North America, where outbreaks predominantly occur in white-tailed deer [31] and mixed livestock/wildlife groups. This cycle differs from outbreaks in grasslands such as ENP and Montana [32]. White-tailed deer are primarily browsers during the anthrax risk period (summer months). Meaning they primarily feed on leaves, soft shoots, or fruits of high growing, woody plants, such as shrubs [33], with little to no grass consumption. So how do white-tailed deer get infected if deer have a low probability of high-dose exposure associated with soil grazing? White-tailed deer first prefer forbs, which are low growing herbaceous flowering plants (a.k.a. weeds) during spring and summer, then in late summer and fall switch to acorns, berries and fruits from browse plants [34]. Summer rain events may increase the forb levels bringing deer into closer contact with low vegetation. These rain events also cause an explosion in biting flies, as was documented during the 2005 epizootic in West Texas [15]. Following feeding on anthrax-infected animals, tabanid horseflies may increase the number of cases through mechanical transmission. It has been shown that *B. anthracis* can be spread to taller vegetation by necrophagous blowflies [35]. After feeding on anthrax carcasses, blowflies land on the leaves of surrounding shrubs, deposit anthrax-containing feces and emesis, thus creating a large three-dimensional infectious zone surrounding the dead animal. The deer then consume the contaminated leaves during browsing near anthrax carcass sites. The cycle of soil to animal to plant to animal serves as a case multiplier and contributes to the epizootiology of anthrax in the white-tailed deer and other exotic animals present across the West-Texas anthrax enzootic area. Termed localized infectious zones (LIZs), these areas surrounding infected carcasses greatly increase the probability of healthy animal exposure. Every West-Texas ranch visited by the authors during the summer of 2019 described a huge explosion in the biting fly population that coincided with the main outbreak wave. Contribution of the LIZ to anthrax transmission was realized by Pasteur in the 19th century [36]. Zebra carcasses in ENP are eviscerated quickly (often within 24 h by nighttime scavengers including hyenas and jackals), but spores persist at the sites for years [25]. The LIZ can be widespread following carcass destruction (out to several meters from the initial death). Flies do not contribute to LIZ development in Etosha because rapid consumption by highly efficient vertebrate scavengers leaves nothing of the carcass for maggot deposition [37]. Horizontal transhumance and migratory animal herding play a role in anthrax outbreaks by concentrating animal density around LIZs and increasing probability of LIZ contact [25,38]. Indeed, if ranch lands in West-Texas are mis-managed, higher than optimal animal densities may drive case numbers higher. The larger a herd that grazes in a high-load anthrax zone the higher the exposure numbers.

With every geography, anthrax infected carcasses deposit a large amount of nutrients in the form of blood and tissue as well as spores. This causes an increase in phosphorous, grass biomass and grass nitrogen levels that correlate with proximity to the zebra carcass site [30]. Other nutrients have been measured but without correlates of distance or adequate non-carcass control sites for comparison, conclusions are uncertain. Vegetative growth and spore load at LIZs have been observed for three years following zebra fall, after which time vegetation and spore levels presumably decrease to background levels. However, during the initial two years following an animal carcass death, *B. anthracis* levels within 1 m of the site remained at ~10^4^ CFU/g of grass, root, or soil. By year three, spores were not detectable on above ground plant biomass but root and soil sample CFUs remained high. During the second year following a carcass fall the increased vegetation appear to increase grazing in the LIZ, perpetuating a low smoldering of anthrax cases. *As we stated above, anthrax epidemics do not occur every year, so increased amounts of vegetation or spores at the LIZ may contribute to outbreak initiation in combination with environmental cues and animal behavior.* The data do not support LIZs alone as a main driver of anthrax epizootics, nor does available data directly consider seasonal rainfall as a major contributor to the process. Because enzootic anthrax often has more than three years between events, it is more likely that seasonal rainfall cycles increase the infectivity of old LIZs by increasing the probability animals come to a former LIZ and when they do, a significant infectious dose is received.

While a wet spring followed by a rain event in a hot dry summer has long been accepted as the prescription for large anthrax events [26,39], few studies have demonstrated this directly, or measured it. In one such study, we used satellite derived NDVI remote sensing data as a proxy for rainfall and discovered the anecdotal evidence do have a firm basis. Earlier vegetation green-up, an earlier start to spring, is correlated with epizootic outbreak years and increased severity of anthrax outbreaks during summers in the Enzootic Zone of West Texas [14]. This zone was defined by our group as a region of frequent large wildlife and livestock outbreaks with evidence of annual cases [15]. For example, over the course of the decade, both 2001 and 2005 were severe anthrax years, where a well-studied white-tailed deer herd in Texas experienced epizootic anthrax outbreaks [14]. Both outbreaks had a statistically significant earlier spring and more intense green-up in the months preceding the large outbreaks. In contrast, enzootic years, those with few or no detected cases, had significantly less green-up and rarely had early green-up [14]. In other words, epizootic years had differences in spring green-up intensity and timing detectable by early April, months ahead of the summer outbreak risk periods.

These observations have added to our evolving knowledge of anthrax and demonstrate that this disease and the way it occurs does not follow the classic textbook descriptions. Further deviation from the textbook is apparent when considering atypical anthrax caused by *Bacillus cereus* biovar *anthracis* (*Bcbva*). This new disease was first described in 2004 and is caused by a strain of *Bacillus cereus* that had acquired toxin and capsule producing plasmids [40]. Anthrax caused by this organism does not appear to have seasonality and predominantly infects non-human primates in the forests of Sub-Saharan Africa, so the epidemiology of wildlife anthrax and anthrax caused by *Bcbva* is also vastly different [41,42,43]. The persistent nature of anthrax in Ta’i National Park represent a problem that current epidemiological models, including those mentioned in this work, do not address.

### 1.2. Modeling Episodic Anthrax

Population dynamics have been used to model single-year anthrax outbreaks [44,45]. Others have taken into account migration of animals into anthrax areas to infer how populations remain persistently susceptible to anthrax [46]. These works are useful in understanding single outbreak population dynamics but become impractical when studying multi-year episodic anthrax outbreaks in areas that do not receive migratory animals from other endemic areas (like mid-latitude anthrax areas in Montana and Texas). Recently, our lab devised a mathematical model considering: (1) environmental cues and (2) host population dynamics to simulate anthrax outbreaks [47]. Without bearing in mind either, the episodic nature of anthrax outbreaks is not sustained with a given influx of anthrax spores. When only seasonal dynamics are included the periodicity of anthrax outbreaks can be obtained but not sustained. When only population dynamics are included, epizootic outbreaks are not observed, and only enzootic disease is simulated. *If both a seasonal driver and population dynamics are included, episodic epizootic anthrax outbreaks are recreated with* in silico *simulations.* By incorporating population dynamics and allowing seasonal forcing of infection to be dependent on an external factor we estimated seasonality to have a large impact on the number of anthrax-related deaths. Thus, environmental drivers coupled with herd population dynamics are required to sustain the episodic nature of epizootic anthrax outbreaks. Our model provides general knowledge of environmentally mediated diseases by explicitly elucidating how intense environmental events determine the tempo and amplitude of outbreaks of rare diseases.

Despite the great differences in infectious cycles, vegetation and geography, some variation of “early wet spring and uncharacteristic heavy rainfall in an otherwise hot-dry summer” appears to hold true across outbreak environments and animal species in enzootic areas. Another important often overlooked initiator of outbreak is the contribution of environmental conditions to population changes of the pathogen and how responses to fluctuating environmental cues affect the ecophysiology of *B. anthracis*. To date, laboratory experiments on the life state of *B. anthracis* have not considered the landscape-level climatic conditions associated with the observed outbreaks and ecophysiology of the pathogen. The availability of our vast and growing global database on outbreaks and environmental conditions across the geography of anthrax [1] allows us the opportunity to define specific laboratory experiments to test such hypotheses like the “wet spring” or “incubator”. The precise mechanisms by which rainfall events induce outbreaks are still unknown. *B. anthracis* spores could be splashed onto nearby lower vegetation where grazers ingest an infectious dose [30]. Simultaneously, during these rain events hydrophobic spores deposited from a previous anthrax carcass may rise to the soil surface as water transitions downwards through the water column or become deposited on plant surfaces during germination of new vegetation. Higher rainfall could also cause shifts in biomass and plant species that increase likelihood of animal contact with soil-borne *B. anthracis* spores. A third provocative explanation may be that rainfall events induce an increase in *B. anthracis* soil populations that then sporulate during dry hot temperatures that follow immediately after.

### 1.3. Soil pH and Calcium Content

Soil conditions that are often concurrent with anthrax outbreaks are increased pH and increased calcium. Anecdotal evidence from outbreak zones around the world suggests elevated pH and calcium levels correlate with enhanced anthrax activity [26,29]. Analysis of soil chemistries in the contiguous United States found several minerals, including calcium, were significantly elevated in counties where zoonotic anthrax cases originated [48]. We hypothesize these two soil characteristics contribute to anthrax outbreaks by enhancing survival of *B. anthracis* through increased sporulation along with mechanical suspension of spores in the soil column. Intact spores bind more efficiently to high calcium soils [49]. It was hypothesized by the authors that positive Ca^2+^ ions in the soil bind to the predominantly negatively charge spore surface, joining the spore to soil particles thus preventing leaching of calcium from the dipicolinate-rich spore. Leaching of calcium can lead to germination failure and loss of spore heat resistance, both very important for bacterial survival in the environment [50,51]. The idea being that the presence of calcium and alkaline pH prevents the spore from being adrift near the inhospitable surface but bound to calcium rich soils deeper in the soil column. Rainfall could allow the buoyant spores to move back up to the soil surface, increasing the probability a grazing host will be inoculated with an infectious dose of spores. Calcium is generally not freely soluble in soils, existing in the form of calcium carbonate (CaCO_3_) or limestone. High calcium soils are often alkaline and have calcium predominantly in the form of calcium carbonate. As calcium carbonate is dissolved by heavy rainfall, free calcium is released and binds to the negatively charged soil particles, bicarbonate is released; the pH rises [52]. As soils dry, the calcium can precipitate as limestone or remain free but immobilized on negatively charged soil particles as a site for spore association with the spore surface serving as a site of calcium nucleation during precipitation [53]. The relationship of soil pH and calcium could have profound effects on the spore but have not been investigated with detail. When an animal dies of anthrax, the bodily fluids are teaming with vegetative bacteria. As the bacteria enter the soil and are exposed to air, they begin to sporulate. The influence of calcium and pH on vegetative cell survival and sporulation rate is not known but could greatly alter the levels of spores that are sustained in an environment. Landscapes of the Texas anthrax enzootic zone are alkaline composites of exposed limestone rock (~40% CaCO_3_) or calciferous loam (~20% CaCO_3_) with limestone rock underneath.

## 2. Methods

### 2.1. Growth and Sporulation of Bacillus Anthracis

*Bacillus anthracis* Sterne 34F2 (Colorado Serum Company, Denver, CO, USA) was grown in BHI broth or agar (Difco Laboratories, Inc, Detroit, MI, USA) and either 30 °C or 37 °C depending on the temperature sensitive nature of the plasmids at use and as described in the manuscript. For sporulation, *B. anthracis* Sterne was grown in BHI overnight at 37 °C with shaking at 250 rpm. The overnight culture was diluted 1:60 in fresh modified G media [54,55] with or without kanamycin at 20 μg/mL then shaken for 72 h at 30 °C and 250 rpm. Cultures were chilled on ice then harvested by centrifugation at 5000× *g* and 4 °C for 15 min in a bench top centrifuge and washed twice with ice cold milli-Q water. Spores were purified from vegetative cells using gradient centrifugation, then incubated in 100% ethanol for 1 h to remove any residual vegetative cells. The spores were washed twice with ice cold milli-Q water then stored at 4 °C until used. Completeness of sporulation was ascertained by microscopy and was typically greater than 99%.

### 2.2. Bacillus Anthracis Growth Experiments

**Figure 2:**
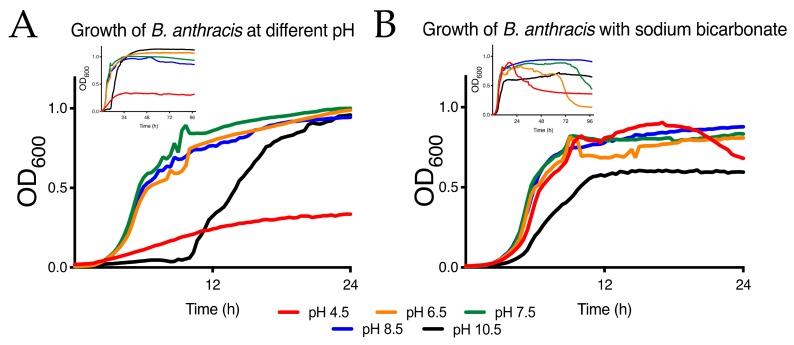
Growth of *B. anthracis* at different pH. (**A**) Short-term and long-term (inset) growth of *B. anthracis* at different pH. Highly acidic pH is especially detrimental to bacterial survival while alkaline pH delays growth but does not impact long-term growth. (**B**) Short-term and long-term (inset) growth of *B. anthracis* at different pH in the presence of bicarbonate. The buffering capacity of bicarbonate is evident by increasing short-term growth rates of *B. anthracis*. Longer term, bacterial survival is optimal at pH 8.5 and 10.5 while optical density sharply decreases at neutral and acidic pH starting at 24 h after growth.

**Figure 3:**
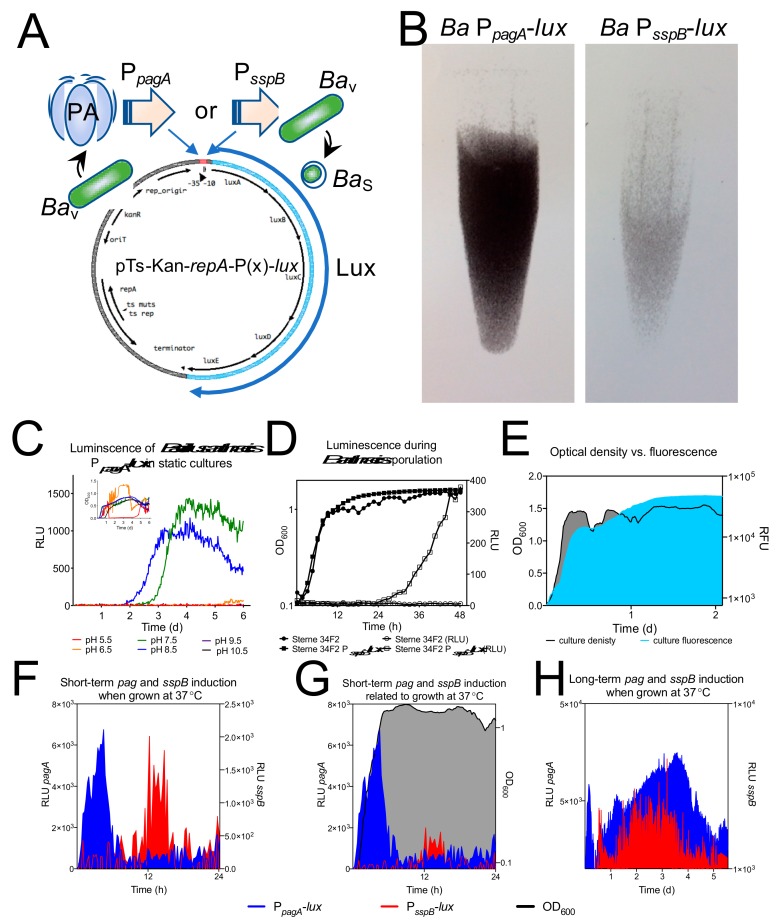
Tracking toxin elaboration and sporulation of *B. anthracis.* (**a**) A diagrammatic representation of the plasmid constructs used to track protective antigen (PA) induction from the *pagA* promoter (P*_pagA_*) in vegetative *B. anthracis* (*Ba*_v_) or sporulation from the *sspB* promoter (P*_sspB_*) in cells preparing to sporulate (*Ba*_s_) at 30 °C. (**b**) A negative luminescent image of cultures grown in liquid broth for 24 h. The strength of expression of P*_pagA_* (left) compared to P*_sspB_*(right) is apparent in this image. (**c**) Static grown cultures of *B. anthracis* at 30 °C show *pagA* expression measured in relative luminescent units (RLUs) is most optimal at pH 8.5 (blue line) and peaks at 3 days. (**d**) Luminescence (clear boxes) induced from P*_sspB_* during shaking growth (black boxes) at 30°C compared to *B. anthracis* luminescence without the plasmid (clear circles) and growth (black circles). (**e**) Validation of our dual-label AmCyan fluorescence indicator of *B. anthracis* growth at 37 °C. Culture density measured by OD_600_ (grey fill) compared to AmCyan relative fluorescent units (RFU; cyan fill). (**f**) Normalized short-term luminescent profiles from P*_pagA_*-*lux* and P*_sspB_*-*lux* expressing *B. anthracis* illustrates the temporally defined stages of toxin production and sporulation. (**g**) P*_pagA_*-*lux* and P*_sspB_*-*lux* expressing *B. anthracis* compared to OD_600_ measurements. (**h**) Long-term luminescence of P*_pagA_*-*lux* and P*_sspB_*-*lux* expressing *B. anthracis* show that after an initial defined period of expression, a second less defined period and then increasing levels of luminescence follow, indicating continued cycles of toxin expression and sporulation in subpopulations of *B. anthracis* in long-term cultures.

Growth experiments were carried out in BHI broth (Difco). For pH experiments, the pH of BHI broth was modified with 1M HCl or 1M NaOH then filter sterilized through a 0.22 µm filter. B. anthracis Sterne was inoculated into pH 7.0 BHI broth and grown overnight at 37 °C with shaking at 250 rpm. The next day, the culture was adjusted to an OD_600_ of 1 in BHI and this was used to inoculate different media at a dilution of 1:100 then aliquoted into clear bottom, black 96 well assay plates and shaken with double orbital shaking at 30 °C on a Synergy H1M microplate reader (BioTek, Winooski, VT, USA). The OD_600_, and luminescence where applicable, of each well were read every 20–30 min for the duration of the experiment (Figure 2A,B and Figure 3C,D). Incubation was the same in static culture except that shaking only occurred for 5 sec before each read. The data shown is the average of three replicates. The standard deviation is omitted from the figures due to clarity. Spores were used to initiate growth assays. Spore preparations were enumerated by dilution plating on BHI and 1 × 10^7^ spore forming units (SFU) were inoculated into each well. Sodium bicarbonate was added at 0.4% (w/v) to growth experiments, as indicated, to investigate the impact of bicarbonate levels on vegetative growth and survival.

### 2.3. Engineering of Bioluminescent B. Anthracis Toxin Elaboration and Sporulation Reporter Strains

Plasmid pMV306hsp+lux was obtained from Addgene [56]. LuxABCDE located on the plasmid was engineered for optimal expression in Gram-positive bacteria by replacement of the ribosomal binding site and reorganization of the genes in the operon [57]. The protective antigen promoter, P_pagA_ was synthesized by GENEWIZ (South Plainfield, NJ, USA) and the small sporulation protein B promoter, P_sspB_, was amplified by PCR and both were cloned via EcoRI/NotI digestion into pMV306hsp+lux replacing the hsp promoter with either the Pag promoter or SspB promoter to create pMV306pag+lux and pMV306sspB+lux, respectively. For maintenance in B. anthracis, the plasmid backbone of pRP1028 [58], which contains the Gram-positive temperature sensitive replicon repA, kanamycin resistance marker and origin of transfer, oriT, was amplified by PCR to introduce NheI and PstI sites then digested with the same enzymes. This fragment was ligated to either P_pagA_ -lux or P_sspB_-lux that were cut from pMV306pag+lux and pMV306sspB+lux with the same enzymes. Clones were verified as bioluminescent. The resultant plasmids were transformed into the mobilizable E. coli strain RHO3 [59] and mated into *B. anthracis* Sterne using standard methods. Luminescent plasmid-containing B. anthracis were selected on BHI agar + 20 µg/mL kanamycin at 30°C and plasmid containing colonies verified by PCR and bioluminescence using a ChemiDoc XRS+ imaging system (Bio-Rad Laboratories, Hercules, CA, USA).

For production of thermostable dual-labeled pRepU-Kan-AmCyan-(P_pagA_ or P_sspB_)-lux plasmids, a PCR fragment from plasmid pRP1099 [58] which encodes the repU B. anthracis replicative plasmid origin, and AmCyan driven by the strong Gram-positive promote, PFP1, was PCR amplified with oligos designed using the NEBuilder online tool (nebuilder.neb.com). This fragment was combined with NotI/PstI digested PpagA-lux or PsspB-lux fragments digested from pMV306pag+lux or pMV306sspB+lux and assembled using NEBuilder HiFi DNA Assembly master mix according to the manufacturer’s recommendations (New England BioLabs, Inc., Ipswich, MA, USA). Fluorescence and luminescence were verified then transferred into *B. anthracis* Sterne 34F2 as described above.

### 2.4. B. Anthracis Growth, Toxin Elaboration and Sporulation Induction Assays in the Presence of Geochemically Relevant Calcium Gradients

Calcium levels in soils of the contiguous United States range from approximately 100 ppm on the low end to an extreme of almost 320,000 ppm [60]. 100 ppm is equivalent to 2.5 mM while the extreme 320,000 ppm is equivalent to 8 M. For growth experiments the range at a low of 1 mM and a maximum of 1 M CaCl_2_ were used to mimic the geochemically relevant gradient of soil calcium concentrations found in soils in the United States. pH was modified with calcium concentrations in a similar manner as in the growth experiments. BHI broth was brought to the desired pH then calcium chloride in MilliQ water was added to achieve the final concentrations. After addition of spores to the growth media suspension, images were captured with a BioRad XR Gel Documentation System. Preparation and growth of the bacteria for these experiments was the same as described above.

## 3. Results

### 3.1. Growth of B. Anthracis at Different pH

pH can vary greatly across soils in a given landscape. The connection between soil pH, anthrax outbreaks and *B. anthracis* recovery from the soil is widely acknowledged but understanding growth of *B. anthracis* and the impact pH can have on the organism using laboratory experiments can lead to interesting findings. In Figure 2, the effect of pH on growth of *B. anthracis* from a spore inoculum is clearly evident. A pH of between 6.5 and 8.5 is ideal for growth of *B. anthracis*, in agreement with the notion that spores are found in more alkaline soils. pH of 4.5 supports meager growth of *B. anthracis*, reaching stationary at an OD_600_ of ~0.3. Growth at higher pH causes a significant lag in growth but then the bacterium reaches the same growth levels when at neutral pH, potentially indicating decreased germination efficiency (Figure 2A). Longer term growth analysis indicates acidic pH is inhibitory to growth of *B. anthracis* (Figure 2A, inset). It is also known that bicarbonate of the human blood pH buffering system can activate virulence genes and impact germination of spores. Bicarbonate is also found in alkaline soils. Calcium ions bind bicarbonate in solution and when water evaporates calcium carbonate (limestone) is inevitably formed. By adding bicarbonate to the growth culture, we can simulate the effect of high moisture levels and alkaline soils on *B. anthracis* survival. 

The short-term (T = 0 to 24 h) growth inhibition of *B. anthracis* at pH 4.5 is essentially eliminated and the extended lag phase at pH 10.5 is greatly reduced (Figure 2B). In the longer-term model (96 h), those gains are not long lived, and bacterial survival is impacted at pH 4.5 to 7.5 (Figure 2B, inset). Survival in pH 8.5 is the only growth not affected by bicarbonate levels.

### 3.2. Tracking Toxin Elaboration and Sporulation Using Bioluminescent Reporter Strains

Static growth or perhaps biofilm formation could allow enhanced growth at otherwise inhibitory pH. To elucidate whether pH can affect induction of anthrax toxin a transcriptional fusion of the bioluminescent *luxABCDE* (abr. *lux)* operon to the protective antigen (PA) encoding gene, *pagA*, promoter was utilized (Figure 3A). PA is the common component of the two anthrax toxins. It binds host cells and translocates either Lethal Factor or Edema Factor into the cell cytoplasm after endocytosis. Together these factors cause cell signaling dysregulation and eventual cell death. The anthrax toxin is a major *B. anthracis* virulence factor so the ability to monitor induction in real-time in various models provides vital information regarding the life state of the pathogen (Figure 3B). To this end, we engineered a plasmid for monitoring *pagA* expression in *B. anthracis.* The Gram-positive optimized *lux* along with a *B. anthracis* compatible origin of replication was used to monitor *pagA* expression using a sensitive bioluminescent plate reader. During static growth in pH 7.5 and 8.5 media *pagA* was strongly induced and, in this model, non-existent at all other pH media tested (Figure 3C). Similar patterns of pH dependent growth were also observed in static *B. anthracis* cultures. In this model, spiking fluctuations of optical density indicate biofilm growth from bacteria growing in close association without shaking (Figure 3C, inset). It is interesting to postulate that association between *B. anthracis* in soil, bacterial survival and toxin expression coinciding at elevated pH may be because of a limited soil lifecycle of *B. anthracis*. pH is just one of many nutrient parameters that could be investigated with this model.

Comparable experiments can be carried out with sporulation reporter strains to elucidate the ecological factors contributing to transition from vegetative *B. anthracis* (*Ba*_V_) to spore (*Ba*_S_) (Figure 3A,B). The spore is a fundamental component of the *B. anthracis* lifecycle and is highly resistant to heat, desiccation, and many antibiocides. Using the small sporulation B (*sspB*) protein promoter upstream of the *lux* gene as reporter, induction of sporulation in different simulated nutrient environments can be directly observed (Figure 3D). As nutrients run low, simulated as stationary phase growth, sporulation proteins are induced, and luminescence rises.

We utilized the *B. anthracis* Sterne 34F2 strain harboring pTs-Kan-*repA*-P*_pagA_*_-_*lux* to follow toxin induction in static culture growth, similar to those used to produce the human AVA vaccine. Alternatively, we have created dual labelled strains that allow fluorescent tracking of *B. anthracis* by tagging with AmCyan fluorescent protein in combination with *pagA* or *sspB* luminescence induction that is not on a temperature sensitive replicon. These plasmids, pRepU-AmCyan-P*_pagA_*_-_*lux* and pRepU-AmCyan-P*_sspB_*-*lux*, were engineered to allow visualization of bacteria within host-cells or thin soil matrices by using AmCyan fluorescent labeling in conjunction with toxin or sporulation luminescence expression tracking. The capacity to track growth of the bacterium with fluorescence was verified by growth curve and a high correlation of fluorescent intensity and optical density of the culture was found (Figure 3E). It can be seen in Figure 3F that short-term expression from the *pagA* and *sspB* promoter occurs sequentially and peak *pagA* expression coincides with exit from exponential growth. The strength (signal is ~ 3 times higher than *sspB*) and timing (expression from P*_sspB_* ensues ~6 h later) of expression from the *pag* promoter is also apparent (Figure 3F,G, respectively). Long-term expression analysis shows that after initial spikes of toxin expression and sporulation expression, both signals increase again, meaning there are subpopulations in the long-term cultures that are out of phase in terms of toxin expression and sporulation (Figure 3H). Microscopy confirmed the presence of vegetative cells and spores in a biofilm like matrix after 6 days (data not shown).

**Figure 4:**
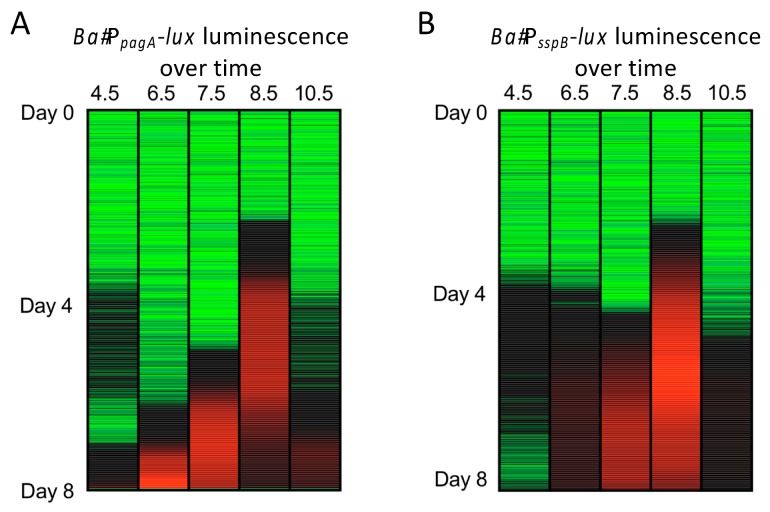
Induction of toxin elaboration and sporulation at different pH are tracked with luminescent reporter constructs. (**A**) Heat-map of luminescence production over time at different pH by P*_pagA_*-*lux* expressing *B. anthracis* when grown with shaking at 30 °C in BHI broth. (**B**) Heat-map of luminescence production over time at different pH by P*_sspB_*-*lux* expressing *B. anthracis* Green = low expression, Red = high expression, black = mid-level expression.

Utilizing our luminescent strains, pH influences *pagA* and *sspB* expression in cultures grown at 30 °C over a week’s time (Figure 4). Expression of *pagA* occurred earliest (~48 h) at a pH of 8.5 followed by pH 7.5. Decreasing pH below 8.5 inhibited expression of *pagA*. At pH 10.5 expression is not observed (Figure 4A). A similar scenario is observed with P*_sspB_*-*lux* expression (Figure 4B). Maximum induction of *sspB* occurs at pH 8.5 and coincides with maximum *pagA* expression. 

### 3.3. pH and Calcium Combine to Modify the Physiology of B. Anthracis

**Figure 5:**
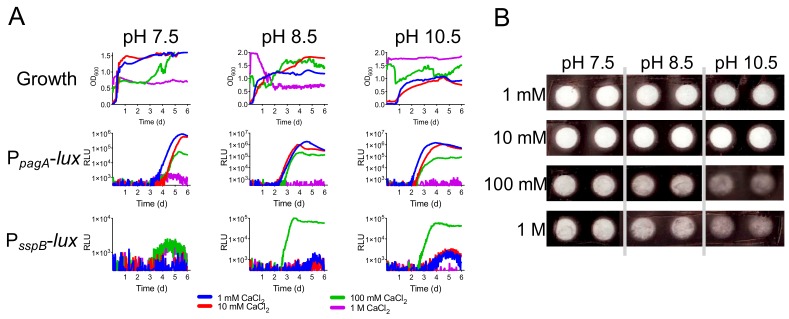
Calcium and pH influence lux expression from P_pagA_ and P_sspB_. (**A**) Long-term growth (top three panels) and luminescence of P_pagA_-lux (middle three panels) and P_sspB_-lux B. anthracis (bottom three panels) across a range of alkaline pH and calcium conentrations. (**B)** Image of precipitation of B. anthracis after addition to pH (vertical) and calcium (horizontal) gradients in 96-well plates at dilution of 1:100 at T = 0. RLU, relative luminescence units.

At pH 7.5 we found that high calcium levels inhibited growth in media while calcium at pH 8.5 and 10.5 restored growth (Figure 5A, top three panels). The highest calcium concntration tested (1 M CaCl_2_) resulted in inhibition of growth. Calcium had minor effects on P_pagA_ induction and expression trends can be attributed to pH. Studies in KNP found Group A *B. anthracis* were found in soils with a mean soil pH of 6.74 and mean calcium content of 185.68 me/kg. Group B strains were found in soils with higher mean soil pH and mean calcium content, pH 7.76 and 274.14 me/kg, respectively. 185.68 me/kg of calcium equals 92.5 mM calcium content and 274.14 me/kg equals 137 mM. These numbers are astoudingly close to our in vitro model where 100 mM CaCl_2_ elicited maximum long-term sporulation at slightly alkaline pH (Figure 5A). 

In Figure 4 we showed that induction of sporulation was inhibited at pH 10.5. In Figure 5A, we show that calcium overcomes this inhibition and 100 mM CaCl_2_ is the optimum concentration tested. Strong induction of lux from P_sspB_ occurs even at the lower concentrations of calcium and earlier compared to induction without calcium. When we added bacteria to the 100 mM and 1 M CaCl_2_ concentrations, bacteria precipitated in the high calcium (Figure 5B). Precipitation occurred in a pH dependent manner. The more alkalaine the pH, the more precipitate formed.

## 4. Discussion

**Figure 6:**
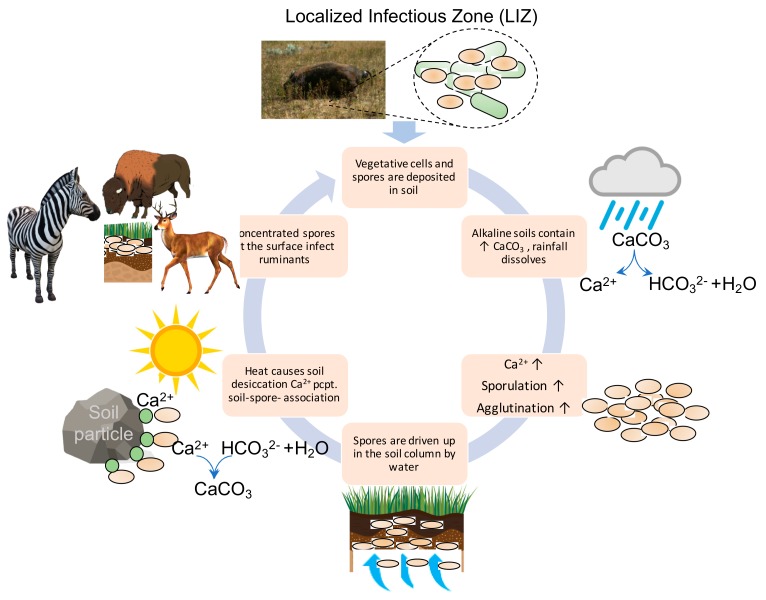
Rainfall, calcium and pH cycling, anthrax microecology and the phenology of anthrax outbreaks. At the top of the cycle, vegetative and sporulated *B. anthracis* are deposited in soil form an anthrax infected animal carcass forming a localized infectious zone (LIZ). Moving clockwise through the figure; if bacteria are deposited in calcium rich alkaline soils, bacterial survival and sporulation are promoted. Rainfall dissolves the mineralized calcium further inducing sporulation and causing bacterial agglutination. Agglutinated spores are likely driven back up through the soil column during regionally heavy rainfall. When hot dry conditions return, desiccation causes spores to attach via ionic interactions to soil particles. Concentrated spores at the surface and reduced food sources during seasonal heat-maxima bring animal hosts into close contact with infectious spore doses. Animals become infected and the cycle continues.

The synthesis of information from our spatial studies and other observations of anthrax outbreaks have shown their cyclical nature on a global scale (Figure 1). This cyclical nature can also be traced to sporadic anthrax outbreaks. Although annual cycles of enzootic anthrax are apparent, the phenology of anthrax epizootics is more complex and is the result of a sporadic cycle [47]. We have been able to differentiate NDVI signatures associated with annual enzootic anthrax and sporadic epizootic anthrax as summarized in Figure 1. However, at the landscape level there must be other forces that perpetuate anthrax endemicity and environmental pathogen load. For example, two different Texas ranches that experience similar annual climatological rainfall events and have strains of similar MLVA type circulating in enzootic years do not experience epizootics at similar rates. Rainfall events drive pH and calcium cycling in soil environments high in limestone and organic matter [61]. (Figure 6). It also helps explain the regional restriction of highly endemic anthrax zones.

These data and other anecdotal evidence led us to investigate pH and calcium influence on *B. anthracis* physiology as a mechanism of anthrax environmental persistence, driven by annual rainfall cycles observed by NDVI remote sensing. Construction and validation of our luminescent reporter strains of *B. anthracis* allowed us to investigate two physiological events vital to *B. anthracis* survival; toxin secretion and sporulation. *B. anthracis* growth, induction of toxin and sporulation are promoted at alkaline pH. Calcium was slightly detrimental to *pagA* expression and very high calcium (1 M) was detrimental to bacterial survival. Toxin is primarily induced during infection in the host, so this outcome was not surprising. In contrast, sporulation was enhanced by nearly 100-fold at alkaline pH in the presence of mid-high calcium levels; which correlated with the measured calcium levels in the highly endemic KNP region. High calcium levels also caused agglutination of *B. anthracis* spores. The geographic and laboratory data has allowed synthesis of a model that better explains the landscape level modification of the ecology of anthrax. 

## 5. Conclusions

We argue identifying global and regional signatures is necessary to fully understand the ecology of anthrax and protect the public health. All over the world, anthrax outbreaks occur with clear periodicity. The phenology of seasonal changes culminates with changing animal behavior to drive anthrax outbreaks following periods of rainfall in a generally hot-dry summer. Figure 1 summarizes data from multiple sources highlighting global seasonal patterns within seemingly sporadic anthrax outbreaks. Regionally, anthrax can be limited to certain areas within landscapes that are experiencing similar weather patterns. Zoonotic outbreaks require the presence of animals but are there environmental components conducive to “hot zone” formation. We know that many types of animals across all continents are involved in anthrax outbreaks, and that hot and dry conditions contribute to outbreaks, whether through animal behavior modification or pathogen ecology alteration. The site of long-term environmental pathogen persistence is soil, thus regional outbreak restriction and chronically contaminated anthrax zones could be due to soil conditions and fluctuation of those soil conditions instigated by rainfall events. pH and calcium have long been linked to increased incidence of anthrax, so we set out in this work to investigate how regional pH and calcium signatures influence zoonotic anthrax disease.

Laboratory experiments can help answer questions the medical geographer may have. For example; what parameters in the soil could increase pathogen burden, persistence, or occurrence? The medical geographer may have information such as pH of soils in a landscape and GPS coordinates of disease or other location specific information that can inform the microbiologist or pathogen ecologist. Currently, medical geographers are analyzing GPS data and ecological factors from the ongoing 2019 West Texas anthrax outbreak. Back in the lab, microbiologists are analyzing fly, plant, and animal material to characterize LIZs, decontamination procedures, and outbreak severity. The information can be used as predictors of risk and impact disease outbreaks of human and animal origin. This work demonstrates how laboratory experiments can help link landscape level drivers, pathogen reservoir modeling, and pathogen physiology in pursuit of a holistic understanding of zoonotic disease outbreaks.
